# Mesh Migration and Bowel Perforation as a Late Complication of Transabdominal Preperitoneal Laparoscopic Hernia Repair

**DOI:** 10.7759/cureus.32683

**Published:** 2022-12-19

**Authors:** Diogo Cardoso, Jorge Rebanda, Catarina Góis

**Affiliations:** 1 General Surgery, Centro Hospitalar de Lisboa Ocidental, Lisbon, PRT; 2 General Surgery, Hospital Dr. Nelio Mendonça, Funchal, PRT

**Keywords:** tack, tack fixation, migration, mesh, inguinal, hernia, tapp

## Abstract

Minimally invasive surgery is increasingly used in the treatment of inguinal hernias, with two main techniques described: transabdominal preperitoneal (TAPP) and totally extraperitoneal (TEP). In both techniques, a prosthetic mesh is placed in a preperitoneal position. However, in TAPP, the peritoneum flap must be completely closed. The TAPP technique is associated with more intra-abdominal complications.

This article describes a case of bowel occlusion due to migration and erosion of a mesh after a TAPP repair in a 57-year-old patient with a history of colonic diverticular disease. The patient complained of abdominal discomfort and constipation, having undergone a colonoscopy and CT scan that demonstrated the presence of a foreign body partially in the lumen of the sigmoid colon. The treatment was surgical, with bowel resection and partial removal of the mesh, complicated by a deep tissue collection. The patient maintained follow-up in a surgery consultation, with no evidence of hernia recurrence.

This is a rare complication of the laparoscopic approach in the treatment of inguinal hernia, more frequent in the TAPP technique. It is intended to draw attention to the type of closure of the peritoneum.

## Introduction

Abdominal wall hernias are very common, of which inguinal hernias account for around 75%. During their lifetime, men have a higher risk of developing inguinal hernias (27%) when compared with women (3%) [1].

Since the 18th century, surgery has been the first option for the treatment of inguinal hernia, with studies showing that each year, more than 20 million hernias are repaired worldwide, with around 750,000 in the United States [2].

Nowadays, there is a wide range of treatments available for the treatment of inguinal hernia, including open primary repair, open tension-free repairs with the use of mesh, laparoscopic repairs with mesh, and watchful waiting. Minimally invasive surgery has become increasingly used in the treatment of inguinal hernias, although the primary indication has been for bilateral and recurrent inguinal hernias. Nowadays, with the acquired expertise, it is already used for the treatment of primary and unilateral hernias. The minimally invasive approach has the potential benefits of shorter postoperative recovery and a decrease in chronic pain [1,3].

The two main techniques described are transabdominal preperitoneal (TAPP) and totally extraperitoneal (TEP). In both techniques, a prosthetic mesh is placed in a preperitoneal position, whereas in TAPP, the peritoneum incision for the placement of the mesh must be completely closed. Although the TAPP technique has a shorter learning curve than TEP, it is associated with more intra-abdominal complications, namely, organ damage, adhesions, or intestinal obstruction [4,5].

## Case presentation

**Figure 1:**
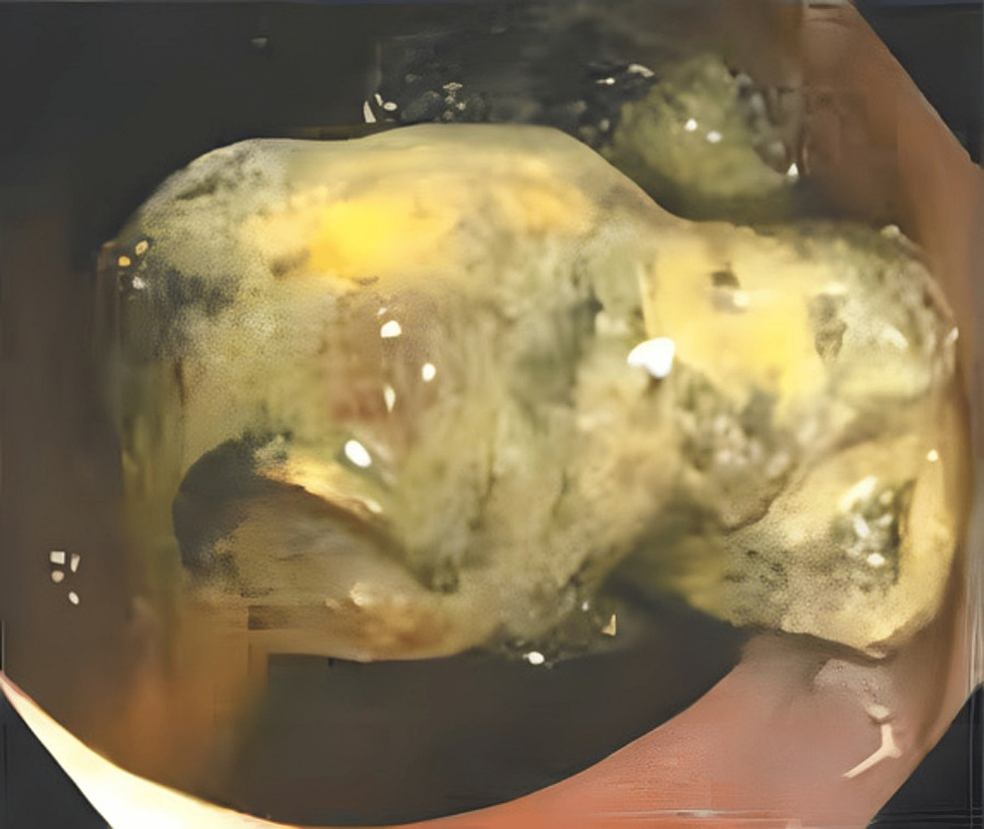
Colonoscopy showing prosthetic mesh with tack

**Figure 2:**
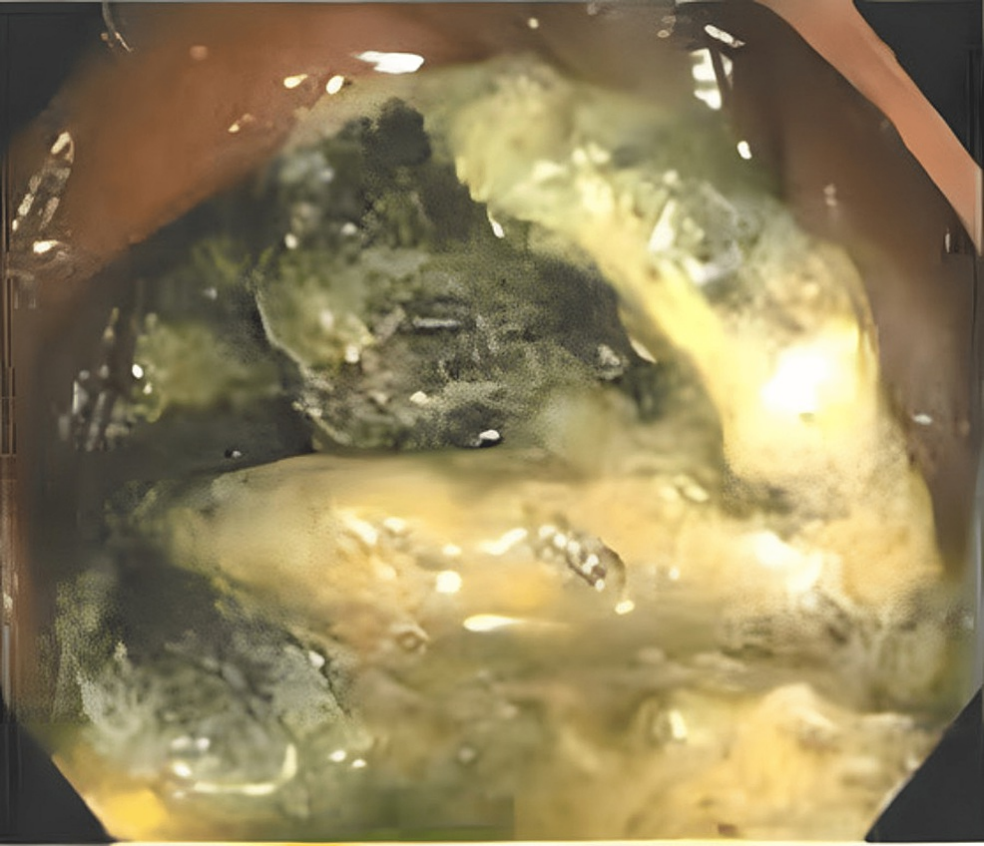
Colonoscopy showing prosthetic mesh occluding the colon lumen

A 57-year-old male patient reported to our outpatient department three years after TAPP bilateral recurrent inguinal hernia repair with a hydrophilic three-dimensional polyester mesh, and closure of the peritoneum with non-absorbable tacks in another institution. Due to complaints of generalized abdominal discomfort and constipation, a colonoscopy was performed, which showed multiple diverticula of the sigmoid colon and a foreign body (at 50 cm from the anal verge) completely occluding the lumen (Figures 1, 2). An unsuccessful endoscopic removal attempt was made.

In our first observation, the patient had no fever, no signs of inflammation in the abdominal wall, regular bowel sounds, and diffuse abdominal pain without signs of peritoneal irritation or palpable masses.

**Figure 3:**
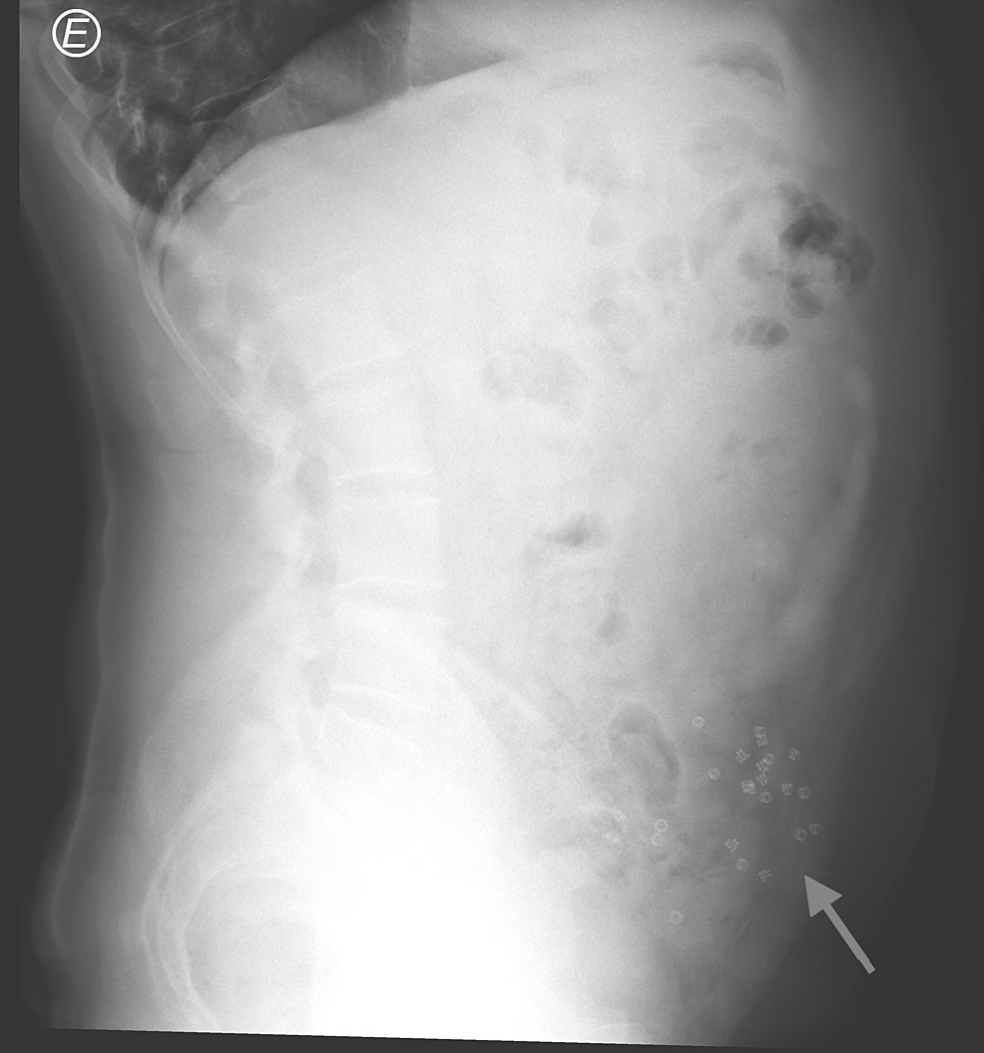
Abdominal X-ray showing multiple tacks (arrow), with an absence of free air or signs of ileus

Routine laboratory investigations showed a slight elevation of C-reactive protein (CRP) and the abdominal X-ray showed multiple spiral tacks in the abdominal wall, with an absence of free air or signs of ileus (Figure 3).

**Figure 4:**
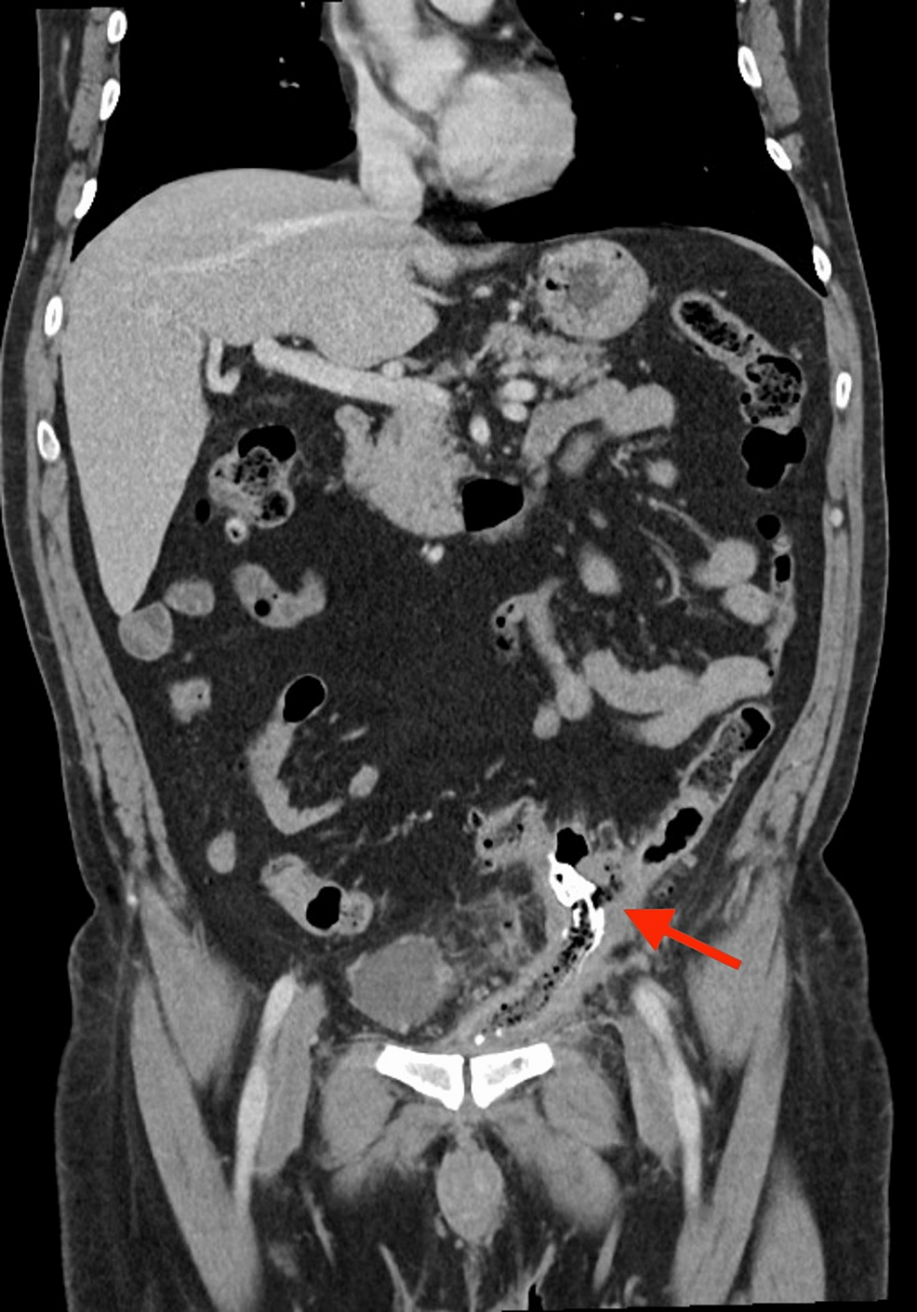
Abdominal CT scan (coronal plane) showing foreign body (arrow) partially in the colon lumen and intraperitoneal collection with air

**Figure 5:**
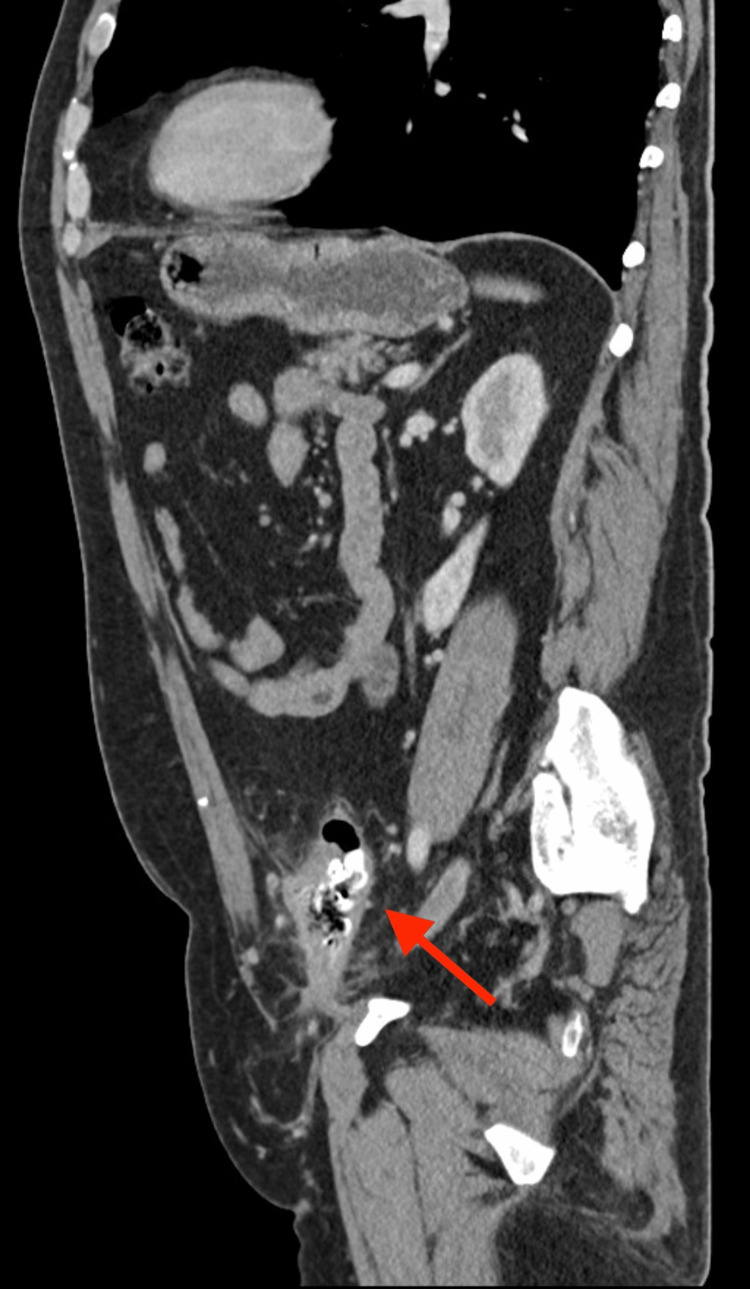
Abdominal CT scan (sagittal plane) showing foreign body (arrow) partially in the colon lumen and intraperitoneal collection with air

In the abdominal computed tomography (CT) scan, a foreign body was evident, partially endoluminal in the sigmoid colon and anteriorly in the Retzius space, associated with an intraperitoneal collection with air (Figures 4, 5).

**Figure 6:**
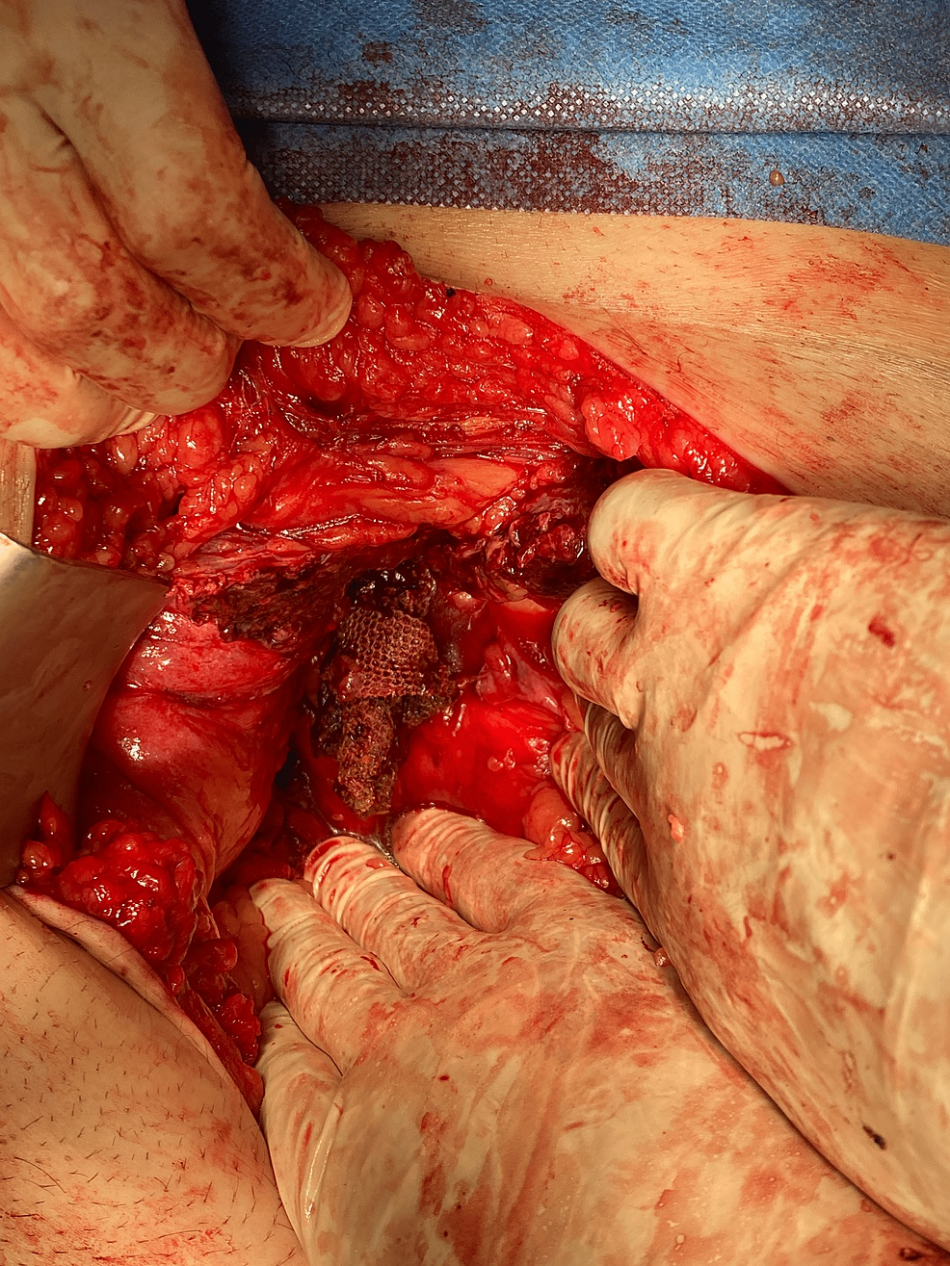
Prosthetic mesh ​​​​​​penetrating the sigmoid colon from the Retzius space

**Figure 7:**
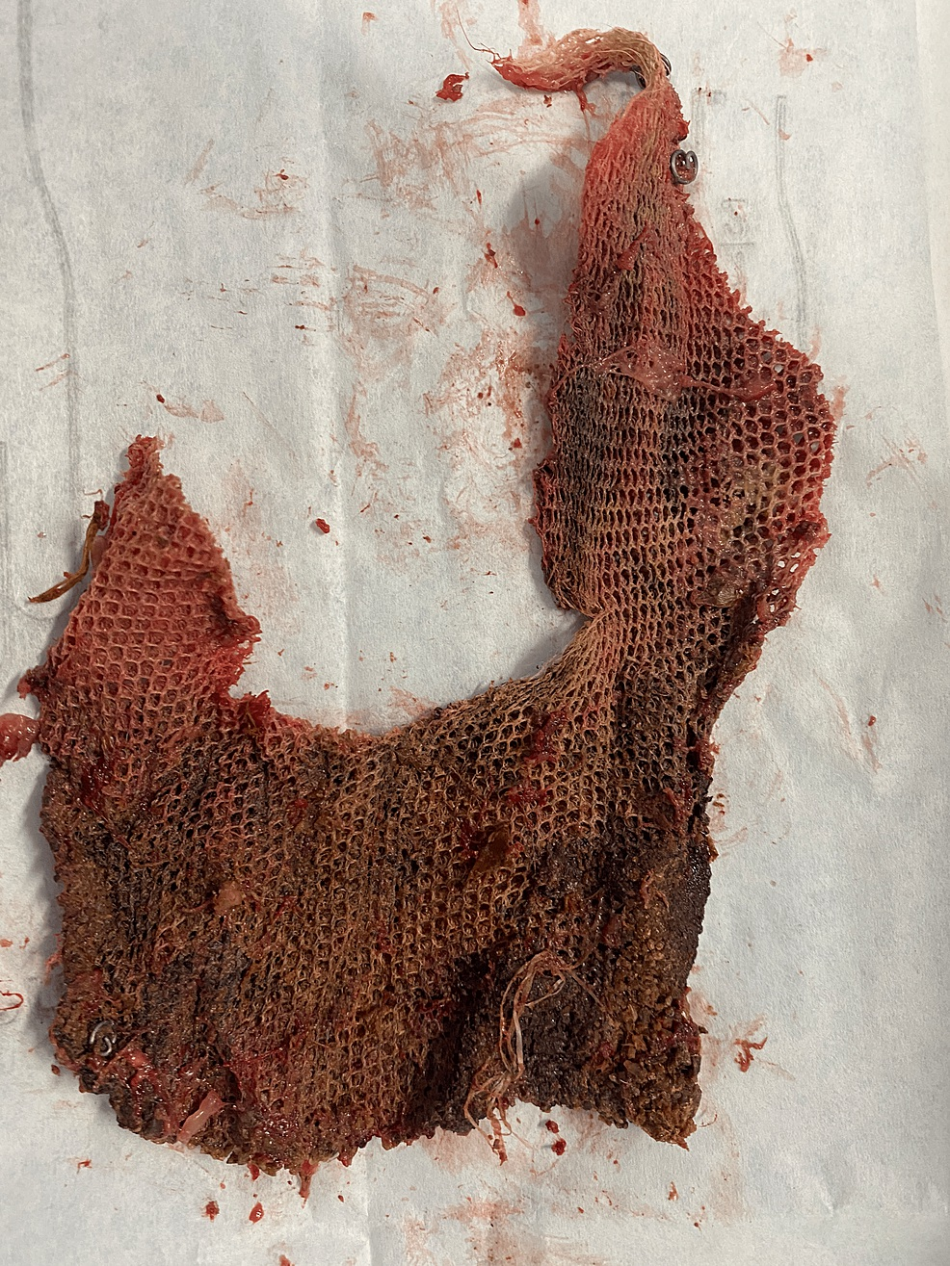
Prosthetic mesh with tacks after removal

**Figure 8:**
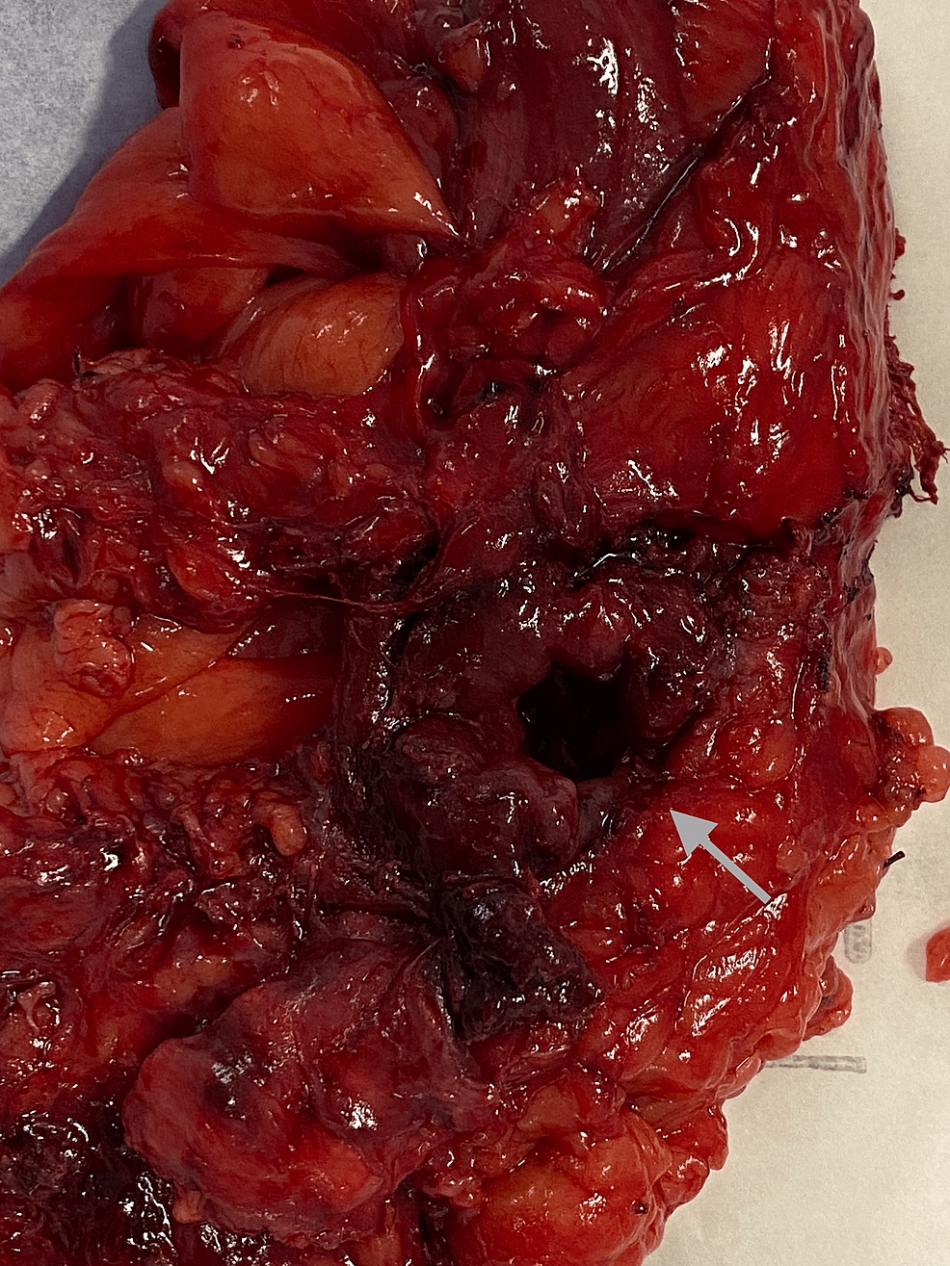
Perforation (arrow) of the colonic wall

The patient underwent exploratory laparotomy, during which a redundant sigmoid colon adherent to the anterior abdominal wall was identified. After its release, a perforation on the colonic wall was found, through which a prosthetic mesh was penetrating from the preperitoneal space (Retzius space) (Figures 6-8).

A sigmoid colon resection with primary anastomosis and partial removal of the mesh from the abdominal wall was performed.

The patient had an uneventful postoperative period and was discharged after eight days on routine oral analgesics. During the routine follow-up (15 days after surgery), he was readmitted due to a deep tissue infection in the Retzius space, treated with ultrasound-guided percutaneous drainage and a course of broad-spectrum antibiotic with piperacillin and tazobactam combination. The patient was discharged 10 days after readmission, maintaining follow-up consultation without hernia recurrence one year after surgery.

## Discussion

Nowadays, there are still many questions surrounding hernia surgery, with not only the indications for surgery but also the best surgical approach that should be used to avoid recurrence or complications and to improve the quality of life. This quest led to the advancement of the use of laparoscopic techniques, which are associated with faster recovery, a better quality of life, and less postoperative pain and wound complications when compared with the traditional open approach techniques. However, the longer learning curve, higher costs, and the potential for more serious visceral and vascular complications are among the disadvantages that have been described [6,7].

When TAPP surgery is performed, there is a need to completely close the peritoneal gap created to access the preperitoneal space. To that end, several methods can be used, such as staples, sutures, tacks, or glue. According to Ross et al., sutured closure of the peritoneum should be the ideal method, allowing a better early postoperative quality of life [8].

The most common way of closure of the peritoneal gap in TAPP repairs is with tacks, mainly because of the ease of the method, which leads to less operative time. However, the use of non-absorbable tacks has been associated with numerous complications, such as chronic pain, adhesions, and intestinal perforation. The use of absorbable tacks and fibrin glue is associated with similar results and fewer long-term complications [5,9].

During their seven-year study of 3017 patients who underwent TAPP, Kapiris et al. described seven cases (0.2%) of small bowel obstruction due to herniation of the small bowel through a defect in the peritoneal closure, with 0.8% occurring when the peritoneum was closed with staples and only 0.1% when the closure was done with suture [10].

Bowel obstruction after TAPP repair is a rare complication. It is usually associated with a deficient closure of the peritoneum, inappropriate fixation of the mesh, adhesions, and hernia formation at the trocar site. An injury to the serosal layer of viscera can be caused either by the edges of the mesh or by the tack, which leads to an intra-abdominal inflammatory process and eventually mesh erosion. Therefore, the importance of adequate closure of the peritoneal flap is well established to prevent mesh contact with the abdominal viscera with fistula formation and mesh migration [5,11,12]. D’Amore et al. suggest that in patients with diverticular disease, episodes of diverticular inflammation could induce the mesh to erode into the colon [13].

The true incidence rates of mesh erosion, migration, and perforation into adjacent organs remain unknown, with the majority of cases reporting the bladder as the affected organ and with clinical manifestations presenting from one to 20 years after the hernia repair [11,12].

The exact cause of the complication described in our case report remains unclear; however, one can argue that it was probably due to the failure to achieve the appropriate closure, allowing the contact of the mesh with the abdominal organs, leading to erosion and mesh migration, resulting in bowel obstruction. Maybe the diverticular disease the patient had and the use of non-absorbable tacks also played a role.

## Conclusions

Currently, laparoscopic TAPP has acquired worldwide popularity and acceptance, being deemed a safe and efficient procedure to treat inguinal hernias. However, it must be remembered that, although rare, some complications can cause significant morbidity. Some of the most dreadful complications described in the literature are the ones that occur due to the fixation techniques of the mesh (staples, tacks, sutures) and the incomplete closure of the peritoneum.

This case reports an avoidable, serious, and potentially fatal complication related to a deficient technique when performing TAPP, highlighting the need for meticulous closure of the peritoneal gap.
